# Soft Tissue Response to Titanium Abutments with Different Surface Treatment: Preliminary Histologic Report of a Randomized Controlled Trial

**DOI:** 10.1155/2016/2952530

**Published:** 2016-06-06

**Authors:** Luigi Canullo, Jan Friedrich Dehner, David Penarrocha, Vittorio Checchi, Annalisa Mazzoni, Lorenzo Breschi

**Affiliations:** ^1^Private Practice, 00198 Rome, Italy; ^2^Department of Oral Surgery, University of Valencia, 46700 Valencia, Spain; ^3^Department of Oral Surgery, Goethe-Universität Frankfurt am Main, 60311 Frankfurt, Germany; ^4^Department of Medical Sciences, University of Trieste, 34129 Trieste, Italy; ^5^Department of Biomedical and Neuromotor Sciences (DIBINEM), University of Bologna, 40100 Bologna, Italy

## Abstract

The aim of this preliminary prospective RCT was to histologically evaluate peri-implant soft tissues around titanium abutments treated using different cleaning methods. Sixteen patients were randomized into three groups: laboratory customized abutments underwent Plasma of Argon treatment (Plasma Group), laboratory customized abutments underwent cleaning by steam (Steam Group), and abutments were used as they came from industry (Control Group). Seven days after the second surgery, soft tissues around abutments were harvested. Samples were histologically analyzed. Soft tissues surrounding Plasma Group abutments predominantly showed diffuse chronic infiltrate, almost no acute infiltrate, with presence of few polymorphonuclear neutrophil granulocytes, and a diffuse presence of collagenization bands. Similarly, in Steam Group, the histological analysis showed a high variability of inflammatory expression factors. Tissues harvested from Control Group showed presence of few neutrophil granulocytes, moderate presence of lymphocytes, and diffuse collagenization bands in some sections, while they showed absence of acute infiltrate in 40% of sections. However, no statistical difference was found among the tested groups for each parameter (*p* > 0.05). Within the limit of the present study, results showed no statistically significant difference concerning inflammation and healing tendency between test and control groups.

## 1. Introduction

Pure titanium (CP-Ti) and titanium alloy Ti-6Al-4V are widely used for dental implant abutments. In fact, titanium and its alloys provide higher strength, rigidity, and ductility compared to other dental alloys. Due to increased esthetic requirements and procedure standardizations, the use of customized instead of prefabricated titanium abutments has been promoted.

Abutment customization can be realized in laboratory or by CAD-CAM procedures. After laboratory customization, microgrooves can be observed on the milled surface of the titanium abutments. In the clinical usage, these microgrooves could result in accumulation of contaminants/debris.

Additionally, contaminants and debris (2–4 *μ* in width microparticles of titanium mixed with lubricant) on the surface and below the finishing line are often detected even after traditional cleaning procedures [1]. Although smoother and cleaner surfaces can be obtained using CAD-CAM procedures, a minimal amount of contaminants is detected after SEM analysis. Additionally, titanium usually exhibits a surface layer of titanium oxide. This surface oxidation together with pollution due to the laboratory workflow results in chemical contamination of the surface, which cannot be eliminated by steam sterilization procedure [2].

Presence of contaminants at the implant platform-abutment level has been suggested to be associated with tissue-damaging inflammation and titanium wear microparticles were demonstrated to activate osteoclastic action [3]. Additionally, according to Albrektsson et al. [4], initial peri-implant marginal bone changes could represent a foreign body response to the implant/abutment complex.

It has been shown how the interactions between cellular components and implant abutment materials influence the stages of the healing process around implants [5]. However, the cells reaction to implanted foreign bodies depends on the material properties, since contaminants and chemical debris could significantly change the composition of the surface, at the interface level with biologic tissues. For this reason, cleaning and disinfection procedures represent a mandatory step during the prosthetic phases of implant-supported rehabilitations.

Few cleansing protocols (through ultrasounds or Plasma of Argon) are proven to allow complete microcontamination removal [6]. Plasma cleaning has been generically defined as a process that uses partially or entirely ionized gas with an approximately equal number of positively and negatively charged particles. Plasma discharge gas is generated by supplying energy to natural gases to form small charge carriers, reactive species, and UV radiations. Low temperature plasmas, used in surfaces modification and organic cleaning, are ionized gases generated at pressures between 0.1 and 2 torr. Low temperature plasmas work within vacuum chambers where atmospheric gases have been evacuated below 0.1 torr. These low pressures allow a relatively long free path of accelerated electrons and ions. At this pressure, the reactions remain at a low temperature. With appropriate plasma parameters, Argon Plasma removes all chemical traces that remain from previous processes.

It has been demonstrated that plasma cleaning has a triple effect on titanium: cleaning, corrosion protection, and surface energy enhancement of the cleaned surfaces. Moreover, cleaning protocols based on Plasma of Argon were proven to allow improved fibroblast adhesion, also suggesting better soft tissue healing around titanium abutments [7].

The aim of this study was to histologically compare the peri-implant soft tissue response to titanium abutments characterized by different surface cleaning treatments. The null hypothesis tested was that Argon Plasma cleaning treatment of the tested abutments did not have any histological effect on the peri-implant tissue inflammation.

## 2. Materials and Methods

A pilot prospective, randomized, controlled trial was performed following the principles outlined in the Declaration of Helsinki. Sixteen consecutive patients were recruited and treated at the Oral Surgery Unit of the University of Valencia (Spain) from July 2011 to December 2012. Any patient older than 18 years, able to sign an informed consent, and in need of tooth replacement in premolar or molar areas of upper and lower jaws was considered eligible for the trial. Reasons for inclusion/exclusion are listed as follows.

*Subject and Study Site Inclusion and Exclusion Criteria*

*Subject Inclusion Criteria*.

Consider the following:

- Need for fixed implant-supported prosthesis in the premolar and molar area in the upper maxilla.
- Age > 18 years.
- No relevant medical conditions.
- Nonsmoker or smoking ≤ 10 cigarettes/day (all pipe or cigar smokers were excluded).
- Plaque Index and bleeding on probing ≤ 25%.
- Presence of a wide ridge of bone allowing the insertion of a 4 mm platform implant according to the Branemark protocol.

*Specific Subject and Site Exclusion Criteria*.

Consider the following:

- Sites with acute infections.
- Pregnant and lactating patients.
- Sites needing horizontal or vertical bone regenerative procedures.
- Patients with a history of bisphosphonates therapy.

All procedures were approved by the local ethical committee of the University of Valencia (H1358503825365). The paper was written following the CONSORT statement for improving the quality of RCTs (http://www.consort-statement.org/).

### 2.1. Surgical Procedures

Each patient was premedicated with 2 gr of amoxycillin and clavulanic acid (Augmentin 1 g, GlaxoSmithKline, Verona, Italy) 1 h prior to implantation. A miniflap with papilla preservation was raised. Using a surgical stent, patients received one implant (Global Implant, 4.8 mm in diameter, Sweden & Martina, Padova, Italy), inserted 0.5 mm below buccal bone level. Immediate impression was taken and, after suturing, implants were allowed for submerged healing.

### 2.2. Prosthetic Procedures

After 3 months of healing, a minimally invasive flap for second surgery procedure was performed and the abutment was screwed at 20 N/cm.

Sixteen screw retained healing abutments, especially designed for the study by CAD-CAM procedures (Echo, Sweden & Martina, Padua, Italy), were randomly divided into 3 groups and allocated to different cleaning processes: (1) Plasma Group: after laboratory customization, abutments underwent Argon Plasma treatment in a plasma reactor (Diener Electronic GmbH, Jettingen, Germany) at 75 W of power and 1 bar of pressure for 12 min; (2) Steam Group: after laboratory customization, abutments underwent cleaning by steam, performed for 5 s at 4 MPa (VAP 1, Zhermark, Cologne, Germany); (3) Control Group: abutments were used as they came from industry and received no further treatment.

### 2.3. Randomization

Immediately before second surgical step, implants included in the study were randomly assigned to one of the three treatment groups. A second operator performed the assignment using sealed envelopes. The envelope was opened at the time of abutment connection and the abutment treatment process related to the selected group was immediately performed chair-side. The surgeon and the patients were blinded to the type of abutment inserted, which was brought by a dental assistant in a sealed envelope.

### 2.4. Biopsy Procedures and Histological Evaluation

Seven days after the second surgery, soft tissues around abutments were dissected using a circular blade of 6 mm in diameter. Immediately after surgery, the 16 soft tissues biopsies were immediately fixed in 4% glutaraldehyde in 0.2 M sodium-cacodylate buffer (Sigma Chemical, St. Louis, MO, USA) at 4°C for 24 h, rinsed in 0.2 M sodium-cacodylate buffer, dehydrated in an ascending series of alcohols in increasing concentrations of ethanol (from 50% to 100%), and embedded in a London White resin (LR White Resin, London Resin, London, England).

After resin polymerization, specimens were sectioned along their longitudinal axes using a high-precision low-speed diamond disk (Micromet, Remet, Casalecchio di Reno, Italy) up to 100 *μ*m thick, glued on glass, and ground to approximately 40 *μ*m thick sections with a specially designed grinding machine (Micromet, Remet, Casalecchio di Reno, Italy) under water irrigation. Ground sections were stained with acid fuchsine and toluidine blue and observed under normal transmitted light using an optical microscope (Nikon Eclipse, Nikon, Tokyo, Japan) at 100x magnification.

On each section, two independent observers assessed the inflammatory expression. Scoring was performed in accordance with a paper by Van Brakel et al. [8]. All prepared full specimens were scored with respect to the degree of inflammation on a 4-point scale. A higher score represents healthier tissues, that is, less inflammatory response: (1) masses of inflammatory cells; (2) many inflammatory cells, showing some fibroblasts; (3) immature connective tissue, showing fibroblasts with few inflammatory cells; (4) normal appearance of connective tissue with few inflammatory cells.

Statistical evaluation of the scores for each parameter was performed using the Kruskal-Wallis nonparametric variance analysis and the probability of the observed outcome or an outcome more extreme is calculated exactly. *α* value indicating statistical difference was* a priori* set at 0.05. Intraexaminer reliability was assessed using the kappa test (*κ*).

## 3. Results

At the end of the study, 16 patients were recruited (9 males and 7 females) presenting a mean age of 62.2 (SD 18.3). One patient was excluded and 15 remained (5 in each group).

Results from the evaluation of the five assessed parameters for each of the three tested groups are reported in Tables 1 [Table tab2] [Table tab3] [Table tab4]–5. The kappa test confirmed the intraexaminer reliability (*κ* = 0.89).

**Figure 1:**
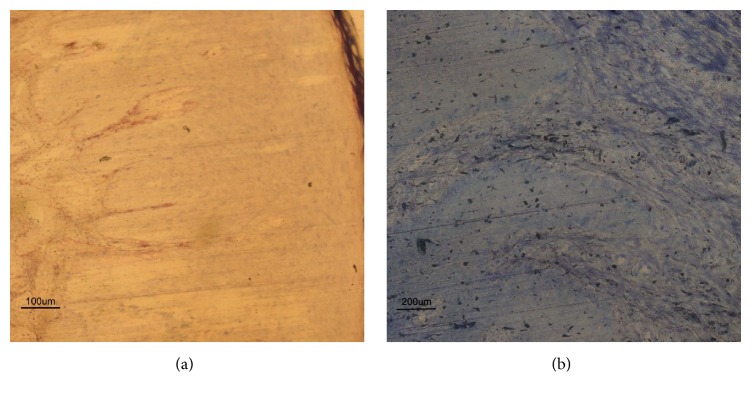
Soft tissue around healing abutment. Histological evaluation of soft tissue surrounding plasma-conditioned abutments (Plasma Group). (a) Histological view showing rete pegs, with little round cell infiltration and to the right keratinized epithelial cells (×100, acid fuchsine and toluidine blue staining). (b) Histological view showing absence of acute infiltrate, minimal chronic infiltration, and diffuse presence of collagenization bands (×200, acid fuchsine and toluidine blue staining).

Histologically, an extreme variability of inflammatory expression factors was found within the sections of each group. Soft tissues surrounding Plasma Group (Figures 1(a) and 1(b)) predominantly showed diffuse chronic infiltrate, almost no acute infiltrate (with presence of few polymorphonuclear neutrophil granulocytes), and a diffuse presence of collagenization bands.

**Figure 2:**
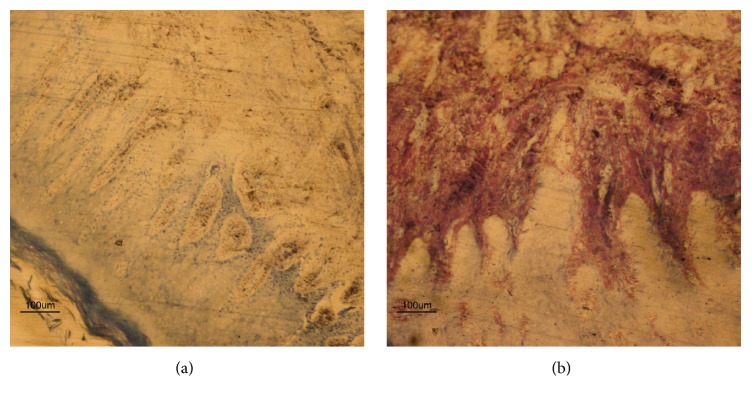
Soft tissue around healing abutment. Histological evaluation of soft tissue surrounding no conditioned abutments, customized by laboratory procedures and cleaned by steam (Steam Group). (a) Low magnification view of a pocket epithelium, revealing minimal acute and chronic infiltrate, diffuse collagenization bands, and pseudoepitheliomatous hyperplasia due to chronic trauma and characterized by multiple layers of keratinocytes with hyper- and orthokeratosis (×100, acid fuchsine and toluidine blue staining). (b) Representative histological image showing absence of acute infiltrate, minimal chronic infiltrate, and presence of collagenization bands (×100, acid fuchsine and toluidine blue staining).

Similarly, in Steam Group (Figures 2(a) and 2(b)), the histological analysis showed a high variability of inflammatory expression factors. Low magnification views revealed minimal acute infiltrate, mild presence of chronic infiltrate, massive presence of collagenization bands, and pseudoepitheliomatous hyperplasia probably due to chronic trauma and characterized by multiple layers of keratinocytes with both hyper- and orthokeratosis.

**Figure 3:**
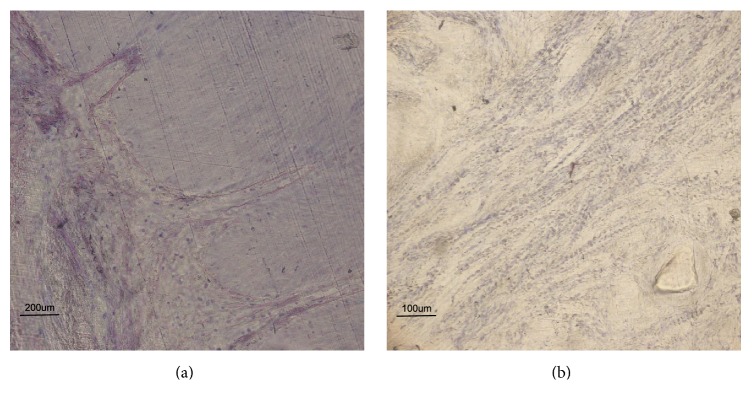
Soft tissue around healing abutment. Histological evaluation of soft tissue surrounding no conditioned abutments (Control Group). (a) High magnification view showing keratinized oral gingival epithelium with rare presence of neutrophils and diffuse presence of lymphocytes and of collagenization bands (×200, acid fuchsine and toluidine blue staining). (b) Low magnification histological view showing keratinized oral gingival epithelium with absence of acute infiltrate, minimal chronic infiltrate, and diffuse collagenization bands (×100, acid fuchsine and toluidine blue staining).

Tissues harvested from Control Group (Figures 3(a) and 3(b), Controls) showed presence of few neutrophils, moderate presence of lymphocytes, and diffuse collagenization bands in some sections, while they showed absence of acute infiltrate in other sections.

All groups showed presence of extraneous material in half of the observed sections.

Despite minimal difference in favor of Plasma Group, no statistical difference was found among the tested groups for each parameter (*p* > 0.05).

## 4. Discussion

Data reported in the present study was focused on the influence of cleaned and noncleaned titanium surfaces and their impact on soft tissues. For long-term implant success, the formation of a soft tissue barrier that prevents bacterial penetration through the transmucosal tunnel is essential. This barrier, facing the abutment, is formed by a fiber rich connective tissue [9] and the quality of this attachment depends on the components in contact with the soft tissues [2]. Presence of contaminants on the abutment surface, such as titanium microparticles, could reduce the adhesion to soft tissues and influence peri-implant tissues inflammatory response [3]. Microstained abutment surfaces are known to cause early inflammatory reactions and various cleaning procedures are helpful to provide healing without complications and to maintain an undamaged and long-lasting soft tissue-abutment interface [10].

The null hypothesis tested was confirmed as histologic results of all groups showed similar inflammatory reactions of soft tissues surrounding the tested abutments, despite minimal differences in favor of Plasma Group.

Although our results, obtained harvesting tissues 7 days after abutment placement, seem not to support one specific cleaning procedure versus another, we can speculate that there is at least a positive short-term effect of cleaned titanium surfaces confirming previous* in vitro* findings. Abutment cleaning procedure using Plasma of Argon increases fibroblast adhesion on titanium surfaces only within the first 8 hours [11].

Unfortunately, limited data are available in the literature concerning the topic of cleaning titanium abutment surfaces using Plasma of Argon. Argon Plasma treatment is able to enhance cell adhesion at titanium surfaces and promotes fibroblast aggregation in early wound healing [12]. Moreover, it has been demonstrated that the treatment of titanium with Argon Plasma is able to reduce titanium oxidative stress, improving its biocompatibility [13].

On the other hand, the effectiveness of cleaning performed in different way (ultraviolet light, steam autoclaving, and Plasma of Argon) has been previously assessed, even if no difference was found by direct comparison between these methods in terms of cell spreading and therefore between the cleaning effectiveness [14].

In a previous histological analysis [15], the performance of different healing abutments after 8 weeks from their placement was evaluated testing hydrophobic machined titanium, chemically modified hydrophilic acid etched titanium, or zirconium alloy. The zirconium abutments showed the highest epithelial and subepithelial connective tissue contact to the abutment surface, and these results led the authors to state that zirconium abutments may have the potential to enhance soft tissue adhesion at the transmucosal aspect of titanium implants [15].

In a recent systematic review, Bishti et al. [16] analyzed the peri-implant tissue response to different abutment materials. After an initial search of 2449 titles, the authors selected only 23 studies after their filtering process. Of these 23 studies, only 4 clinically evaluated the peri-implant soft tissue reactions to different abutments assaying bleeding on probing [17], gingival inflammation [18], amount of keratinized gingiva around abutments, and gingival recession [19], while no histological parameter was analyzed.

In the present study, soft tissues response to the differently treated abutments was assayed by quantitative histological analysis, rather than qualitative one as in previous reports. However, despite the improvement in the analysis, no difference was found among the tested cleaning procedures.

The same quantitative approach previously allowed comparing the histological responses of peri-implant tissues in switching and traditional platform implants after 4 years from restoration showing differences in percentage of inflammatory infiltrated area and collagen content [20].

Van Brakel et al. [8] compared the health of soft tissues towards zirconia and titanium abutments, obtaining biopsies after 3 months from abutment placement. Histologic analysis showed little signs of inflammation and well-keratinized stratified squamous epithelium facing the abutment surface [8]. Conversely, soft tissue biopsies included in our study have been harvested after 7 days since abutments placement. Probably, the strength and process of the inflammatory reaction could change harvesting the tissue with an interval of one or more consecutive weeks of healing [21].

Additionally, to better characterize the healing and inflammation process around abutments cleaned with different procedures, additional studies are currently ongoing to perform a quantitative immunohistochemical analysis of tissue fragments and evaluate presence and amount of Vascular Endothelial Growth Factor (VEGF) in keratinized gingiva around healthy and failing dental implants [22].

In conclusion, the results of the present study showed no improvement with plasma cleaning treatment of titanium abutments if compared to steam cleaning or even to untreated abutments, as no difference was found between the tested groups for inflammation and healing around the implants. Future studies should allow defining cleaning procedures to achieve surface disinfection of prefabricated and custom-made titanium surfaces that would allow forming a barrier to prevent bacterial penetration through the mucosal tunnel.

Additional randomized controlled clinical trials are currently ongoing associated with further immunohistochemical analyses, with a multicenter approach, to confirm the effectiveness of the cleaning disinfection protocol on the healing of soft tissues around plasma-treated titanium abutments.

## 5. Conclusions

Within its limits (small sample size and long healing period), results of this preliminary study showed no statistically significant difference concerning inflammation and healing tendency after 1 week between test and control groups. Introduction of cleaning procedures based on Plasma of Argon or steam was not proven to be successful for soft tissue healing.

However, bigger effort in the research of soft tissue response in early wound healing stage appears strictly recommendable.

## Figures and Tables

**Table 1:** Report of the histological analysis of acute infiltrate (exact significance: *p* = 0.734; point probability: *p* = 0.133).

	Score	% sections
Group 1	1	0%
	2	20%
	3	20%
	4	60%

Group 2	1	0%
	2	0%
	3	40%
	4	60%

Group 3	1	20%
	2	0%
	3	40%
	4	40%

**Table 2 tab2:** Report of the histological analysis of chronic infiltrate (exact significance: *p* = 0.500; point probability: *p* = 0.150).

	Score	% sections
Group 1	1	40%
	2	20%
	3	40%
	4	0%

Group 2	1	20%
	2	0%
	3	80%
	4	0%

Group 3	1	20%
	2	0%
	3	80%
	4	0%

**Table 3 tab3:** Report of the histological analysis of collagenization bands (exact significance: *p* = 0.441; point probability: *p* = 0.053).

	Score	% sections
Group 1	1	40%
	2	0%
	3	60%
	4	0%

Group 2	1	60%
	2	40%
	3	0%
	4	0%

Group 3	1	60%
	2	20%
	3	20%
	4	0%

**Table 4 tab4:** Report of the histological analysis of foreign material (exact significance: *p* = 0.500; point probability: *p* = 0.250).

	Score	% sections
Group 1	1	0%
	2	60%
	3	0%
	4	40%

Group 2	1	0%
	2	20%
	3	0%
	4	80%

Group 3	1	0%
	2	20%
	3	0%
	4	80%

**Table 5:** Report of the histological analysis of pseudoepitheliomatous hyperplasia (exact significance: *p* = 0.301; point probability: *p* = 0.100).

	Score	% sections
Group 1	1	40%
	2	0%
	3	0%
	4	60%

Group 2	1	60%
	2	0%
	3	0%
	4	40%

Group 3	1	0%
	2	20%
	3	0%
	4	80%
